# Identification, pathogenicity, and virulence of the causal agent of bacterial scab disease on mango in China

**DOI:** 10.1186/s12866-025-04656-3

**Published:** 2025-12-22

**Authors:** Feng Liu, Yanling Xie, Fangli Hu, Naihui Lu, Hong He, Rulin Zhan, Quansheng Yao, Guizhen Li

**Affiliations:** 1 South Subtropical Crops Research Institute, Key Laboratory of Hainan Province for Postharvest Physiology and Technology of Tropical Horticultural Products, Key Laboratory of Tropical Fruit Biology, Chinese Academy of Tropical Agricultural Sciences, Ministry of Agriculture, Zhanjiang, 524013 China; 2 Agricultural and Forestry Research Institute of Panzhihua, Panzhihua, 617061 China

**Keywords:** Mango bacterial scab desease, Isolation and classification, Enterobacter hormaechei, Pantoea disperse, Chemical agents screening

## Abstract

**Background:**

Mango is an important economic fruit in China. As the mango cultivation areas continue to expand, symptoms of bacterial scab disease have been observed on mango fruits from orchards in Hainan Province, China.

**Results:**

Through a combination of morphological diagnoses, pathogenicity tests, physiological and biochemical characterizations, and phylogenetic analysis, the causal pathogens were identified as three distinct bacterial species: *Enterobacter hormaechei*, *Pantoea dispersa*, and *Pseudomonas oryzihabitans*. When compared to *Xanthomonas citri* pv. *mangiferaeindicae* XC01, the causal agent of bacterial black spot in mangoes, *E*. *hormaechei* produced more cell wall degrading enzymes (CWDEs), while *P. disperse* produced more cellulases but fewer proteases. Moreover, these two strains released higher amounts of extracellular polysaccharides (EPS). In contrast, *P*. *orzihabitans* could not produce cellulases or proteases, except for amylases. Additionally, we evaluated the motility capabilities of the three strains, including swimming and swarming motility. All strains showed lower swimming abilities than XC01. Specifically, *E*. *hormaechei* and *P. disperse* swam more slowly than XC01, and only *P*. *disperse* exhibited faster swarming behavior compared to XC01. Among the twelve tested chemical agents, albendazole showed the highest inhibition rates against *E*. *hormaechei* and *P*. *disperse*, with EC_50_ values (the concentration required for 50% maximal effect) recorded at 0.0002 mg/L and 0 0.0001 mg/L, respectively.

**Conclusions:**

This study is the first to identify *E*. *hormaechei*, *P*. *dispersa*, and *P*. *oryzihabitans* as new pathogens of mango bacterial scab disease. These findings lay a foundation for future field control strategies against mango bacterial scab.

**Supplementary Information:**

The online version contains supplementary material available at 10.1186/s12866-025-04656-3.

## Background

 The mango tree (*Mangifera indica* L.) is widely distributed in numerous tropical and subtropical regions. India stands as the world’s leading mango producer, accounting for 38.19% of the global output, followed by China (18.4%), Thailand (6.7%), Indonesia (4.3%), and Mexico (4.3%) [1]. Mangoes are not only significant sources of nutrients; mango production also serves as a crucial livelihood for local farmers in countries such as Kenya and India [2, 3]. Moreover, mangoes are widely cultivated across southern China, particularly in the provinces of Guangxi, Hainan, Yunnan, Sichuan, and Taiwan, and they are regarded as one of the most important tropical fruits [4].

Mango fruits are highly susceptible to various pathogens during their growth periods, which impedes the development of mango trees and results in yield reduction. Anthracnose, caused by *Colletotrichum gloeosporioides*, is one of the most severe diseases affecting mangoes, leading to substantial post-harvest decay [5]. Other important diseases that affect mango fruits and leaves mainly include *Pseudoidium anacardia* (Powdery mildew), *Lasiodiplodia theobromae* (Stem-end rot), *Pseudomonas syringae* pv. *syringae* (bacterial apical necrosis), *Xanthomonas campestris* pv. *mangiferaeindicae* (Bacterial leaf spot) [6–9]. The symptoms of bacterial leaf spot initially manifest as small water-soaked spots at the lenticels. Subsequently, these spots evolve into star-shaped, eruptive lesions that secrete an infectious gum, resulting in characteristic tear-stain patterns. In instances of severe infection, extensive defoliation and/or premature fruit drop may occur. Mango scab, caused by the fungus *Elsinoë mangiferae*, is typified by the development of diminutive, black anthracnose-like lesions on young fruits. This condition frequently leads to substantial fruit drop [10]. Additionally, species like *Pantoea agglomerans*, *Pantoea ananatis*, and *P*. *vagans* are capable of inducing necrotic symptoms in mango trees [8, 11]– [12]. In 2020 and 2021, symptoms of black-brown spots resembling those of bacterial black spot were observed in Sanya, Hainan, China, and the incidence rate ranged from 10% to 25%. As the disease progressed, scab symptoms emerged, which were characterized by the absence of gum flow, distinguishing them from bacterial black spot. The objectives of this study were to isolate and identify the pathogen causing bacterial scab disease.

The *Enterobacteriaceae* family consists of Gram-negative bacteria ubiquitously present in agricultural and natural ecosystems. Certain genera within this family frequently responsible for devastating plant diseases, leading to substantial reductions in crop yields. These predominant genera mainly encompass *Brenneria*, *Pantoea*, *Dickeya*, *Erwinia*, *Lonsdalea*, and *Enterobacter*. Members of the *Enterobacteriaceae* family have been detected in post-harvest strawberry fruits [13]. *Dickeya zeae* is acknowledged as the causative agent of foot rot diseases in maize and rice. Furthermore, it has also been identified as a pathogen of banana plants, causing severe economic losses in China [14]. Fire blight of pome trees, caused by *Erwinia amylovora*, brings about significant losses across the entire value chains of the affected fruits [15].

Moreover, various *Enterobacter* species have shown their pathogenic potential against different hosts. For example, *Enterobacter cloacae* causes bulb decay in garlic; *Enterobacter mori* is associated with bacterial wilt in strawberries; *E. cloacae* and *Enterobacter roggenkampii* are linked to pith necrosis in tomatoes; and *Enterobacter kobei* is implicated in flag-leaf sheath spot disease on rice [16–19]. Additionally, *Pantoea* species also have diverse impacts. *Pantoea agglomerans* leads to black spots on walnuts; *Enterobacter asburiae* and *Pantoea ananatis* cause bacterial blight on rice; and *Pantoea anthophila* is associated with soft rot disease on wampee [20–22].

The genus *Pseudomonas* includes various plant pathogens affecting numerous economically important crops. Main ones are *Pseudomonas savastanoi*, *Pseudomonas cichorii*, *Pseudomonas fragariae*, *Pseudomonas boreofloridensis* and *Pseudomonas citrulli*, causing leaf spots or even rot in lettuce, tomato, strawberry, and watermelon plants [23–26].

Currently, extensive screening and application of fungicides and chemical agents are underway for controlling mango bacterial diseases. Key agents include copper fungicides, benzimidazole fungicides, and antibiotic fungicides [27]. In a laboratory assessment of 22 chemical agents against mango bacterial black spot, tetramycin, octylamine acetate, bromothalonil, and benziothiazolinone showed strong bacteriostatic activity. A a 2 mg/L treatment, the inhibitory rate reached 90%. Pothioconazole and zhongsengmycin followed, with up to 75% inhibitory at 10 mg/L [27]. Previous research indicated that fludioxonil and cyprodinil effectively reduce the incidence of mango stem-end rot [28]. Haituk et al. [29] reported carbendazim, trifloxystrobin, mancozeb, and prochloraz could inhibit the mycelial growth of *Pseudoplagiostoma mangiferae*. In Brazil, early-stage application of mancozeb controlled maize white spot disease (*P. ananatis*) [30]. These findings suggest that fungicides and chemical agents hold potential for preventing and controlling mango bacterial scab.

In this study, black-brown spot symptoms were observed on mango fruits in Hainan Province. Pathogenicity verification, molecular identification, phylogenetic analysis, and physiological and biochemical tests were systematically carried out on the pathogenic strain isolated from scabby mango fruits. Additionally, virulence factors such as cell wall degrading enzymes (CWDEs), extracellular polysaccharides (EPS), and cell motility were investigated. The efficacy of twelve chemical agents against the pathogenic strain was also evaluated to identify effective treatments for this disease. This study identified *E*. *hormaeche*, *P. dispersa* and *P*. *oryzihabitans* as novel pathogens of mango bacterial scab disease.

## Materials and methods

### Sample collection, isolation and purification of pathogens

In 2020 and 2021, samples exhibiting scab symptoms were collected from Sanya, Hainan Province. The infected fruits were gently washed with tap water and subsequently dried. Samples with typical diseased spots were selected and cut into small pieces (5 × 5 mm) along the lesion edges. The surfaces were disinfected with 75% alcohol for 30 s, followed by treatment with a 3% sodium hypochlorite solution for 2 min. Subsequently, the samples were rinsed three times with sterile water and dried on sterile filter paper. Thereafter, the samples were placed in 1 mL of distilled water (SDW) in a sterile grinding dish. A grinding rod was employed to homogenize the diseased tissue. The homogenate was incubated at room temperature for 15 min before plating aliquots of 100 µL and performing ten-fold serial dilutions onto nutrient agar (NA) medium. The NA medium consisted of 5 g/L beef extract, 10 g/L peptone, 5 g/L NaCl, and 15 g/L agar. The plates were incubated at 28 °C for 48 h, and this process was repeated three times as described by Schaad et al. [31].

Isolates with distinct colonial morphologies were selected for further investigation. These strains were cultured in Luria-Bertani (LB) broth medium (comprising 10 g/L tryptone, 5 g/L yeast extract, 10 g/L NaCl) and then evaluated on mango leaves and fruits using the methods described below.

### Pathogenicity tests

To determine the pathogenicity of these strains on mango leaves and fruits, two distinct pathogenicity tests were performed to fulfill Koch’s postulates [8].

Firstly, in-vitro pathogenicity tests were conducted on mango fruits. Selected mango fruits were immersed in 70% ethanol for 30 s, then rinsed three times with sterile water, and subsequently placed on sterile paper to dry. Using a sterile needle, scratches were made on the mango fruits before inoculating them with 10 µL of the diluted bacterial solution (10^8^ CFU/mL). These specimens were covered with moist sterilized cotton and placed in plastic boxes sealed with plastic wrap to maintain humidity. For each isolate, the experiment involved eight fruit specimens and was replicated three times. In the control group, an equal volume of sterile water was applied to the fruits. The plastic boxes were kept in a biological incubator at 28 ± 2 °C, with a relative humidity of 95% ± 15% under dark conditions. Symptoms were observed after seven days. Pathogenicity was determined by observing and measuring the lesion length on the fruits. Three representative strains isolated from mango (MG-1, MG-2, MG-3) were selected. At the end of the experiment, re-isolation and identification of the causal agent were performed through morphological characterization and DNA sequence comparisons.

Secondly, Koch’s postulates were verified using thirty 2-year-old potted mango plants. The leaves of these plants were infiltrated with a diluted bacterial solution (10^8^ CFU/mL) using a sterile needleless syringe. Both sides of the leaf veins on the abaxial surface of the leaves were treated symmetrically, leading to the appearance of visible water-soaked spots. Sterile water was injected as a negative control. All plants were kept in a greenhouse at a temperature of 28 ± 2 °C and a relative humidity of 75% ± 15%. They were watered with sterile water twice weekly until disease symptoms emerged. Pathogenicity was assessed by observing and measuring the lesion length on the leaves after 15 days. Three representative strains (MG-1, MG-2, MG-3) were selected from those used in the previous in-vitro test on mango fruits. At the end of the experiment, re-isolation and identification of the causal agent were performed through morphological characterization and DNA sequence comparisons.

### Molecular identification and phylogenetic tree

Bacterial strains were cultured in LB medium and incubated overnight at 28 °C with shaking at 180 rpm. A 1.5-mL aliquot of the bacterial culture was centrifuged, and the supernatant was discarded to obtain the bacterial pellet. DNA extraction from the pathogenic strain was performed using the TIANamp Bacteria DNA Kit (TIANGEN Biotech CO., Ltd., Beijing, China), following the manufacturer’s instructions. The extracted DNA samples were stored at −20 °C for subsequent analysis. The gene sequences of 16 S rDNA, *rpoB* (RNA polymerase β subunit), *fusA* (elongation factor G) and *gyrB* (DNA gyrase subunit B) were amplified using the primers listed in Table S1 [32–36].

The PCR amplification reaction system had a total volume of 25 µL, which included 1µL of DNA template, 1µL each of forward and reverse primers, 9.5 µL of ddH_2_O, and 12.5 µL of 2 × Phanta Max Master Mix (Vazyme Biotech Co., Ltd., Nanjing, China). The reaction conditions were as follows: an initial denaturation step at 95 °C for 3 min; followed by 30 cycles of denaturation at 95 °C for 10 s, annealing at a suitable temperature for 30 s (Table S1), and extension at 72 °C for 30 s; a final extension step at 72 °C for 7 min. The PCR products were analyzed by electrophoresis on a 1.0% agarose gel and then subjected to Sanger sequencing (Huada Gene Technology Co., Ltd., Shenzhen, China). The obtained DNA sequences were submitted to the National Center for Biotechnology Information (NCBI), and the accession numbers indicated in Table 2.

To clarify the evolutionary status of isolates MG-1, MG-2, and MG-3, phylogenetic analyses were performed based on the sequences of 16 S rDNA, *rpoB*,* fusA*, and *gyrB* genes from these isolates and their closely related strains downloaded from the NCBI database after a BLASTn search (Table 2, Table S2). Phylogenetic trees were constructed using maximum-likelihood clustering analysis implemented in MEGA 6 software [37].

### Morphological, physiological and biochemical characteristics

Strains MG-1, MG-2, and MG-3 were examined under a 100 × oil immersion lens using a photon microscope (Leica Microsystems, Wetzlar, Germany) and were subjected to Gram staining following the manufacturer’s instructions (Solarbio Science & Technology Co., Ltd., Beijing, China). The identification of strains MG-1, MG-2, and MG-3 was carried out through physiological and biochemical tests according to the manufacturer’s instructions (Qingdao Hope Bio-Technology Co., Ltd., Qingdao, China) and “The Laboratory Guide for Identification of Plant Pathogenic Bacteria” [31].

### Measurement of extracellular enzyme activities

To elucidate the virulence factors produced by strains MG-1, MG-2, and MG-3, the activities of cellulases (Cel), proteases (Prt) and amylases (Amy) were assessed. First, the media for semiquantitative assays of enzymatic activities were prepared according to previously described recipes with some modifications (Table S3) [20, 38]– [39]. Then, 15 mL of the medium was poured into each 9-cm-diameter round plate. After the medium solidified, 5-mm-diameter wells were punched, and 20 µL of bacterial cells (OD_600_ = 1.0) were added into these wells. All plates were incubated at 28 °C for 48 h. Then, the diameters of the zones surrounding the inoculation sites were, measured. For the cellulose assay plate, it was stained with a 0.1% (wt/vol) Congo red solution and then treated twice with a saturated NaCl solution. Transparent halos around the wells on the protease assay plate indicated protease activity. For the assessment of amylase activity, the assay plate was stained with a diluted I_2_/KI solution (a1:100 ratio of 0.08% mol/L I_2_ and 3.2 mol/L KI) for 10 min. Each experiment was conducted in triplicate.

### Asssay of EPS production

The production of EPS by isolates MG-1, MG-2, and MG-3 was measured following the procedures described in previous studies with minor modifications [16]. Single colonies were selected from LB agar plates and cultured overnight in LB broth medium at 28 °C with shaking until the optical density at 600 nm (OD_600_) reached 1.8. Subsequently, 20 mL of this culture was transferred to 200 mL of fresh LB medium and incubated for an additional 48 h at 28 °C with shaking at 180 rpm. After incubation, 50 mL of the cultured medium was sampled and centrifuged at 5000 rpm for 40 min to obtain the supernatant. Absolute ethanol was added to the supernatant at a volume ratio of 1:3. After thorough mixing, the mixture was left at 4 °C for 12 h to allow for precipitation. Subsequently, the mixture was centrifuged at 5000 rpm for 40 min to obtain the precipitate. The precipitate was dried at 55 °C overnight and then weighed. Each experiment was replicated three times.

### Measurement of cell motility

To evaluate the cell motility, the media for swimming and swarming assays were prepared (refer to Table S3). A 1-µL aliquot of each strain (OD_600_ = 1.0) was inoculated onto plates filled with 15 mL of the medium. The plates were then incubated at 28 °C for 3 days, after which the diameters of the bacterial motility zones were measured. This experiment was carried out in triplicate [40].

### Chemical agent sensitivity test

The inhibitory effects of twelve chemical agents against isolates MG-1 and MG-2 were assessed using the growth rate method. Different fungicides were dissolved in water (Table S4). Each fungicide was diluted into three concentration gradients as detailed in Table S4. A total of 5 mL of bacterial culture (OD_600_ = 1.0) was added to 50 mL of NA medium. After thorough mixing, the mixture was poured into 9-cm-diameter round plates and allowed to cool until solidified. Subsequently, 5-cm-diameter wells were punched out and filled with 50 µL of each fungicide solution, whereas an equal volume of water was used as the control group (CK). The inoculated plates were placed in a constant temperature incubator set at 28 °C for 48 h. The percent inhibition was determined by measuring the transparent halos surrounding the wells. The EC_50_ values, which represent the concentrations that achieve 50% efficacy, were calculated through toxicity regression analysis using DPS V7.05 software [41]. Each treatment condition was replicated three times.

### Data analysis

One-way and two-way analysis of variance (ANOVA) were employed for data analysis, and all graphical representations were generated using GraphPad Prism version 6.01 (GraphPad Software, San Diego, CA, USA). The statistical significance of the differences between means was evaluated using Dunnett’s test and Tukey’s test at a significance level of *p* ≤ 0.05.

## Results

### Field observation and disease symptoms

**Fig. 1:**
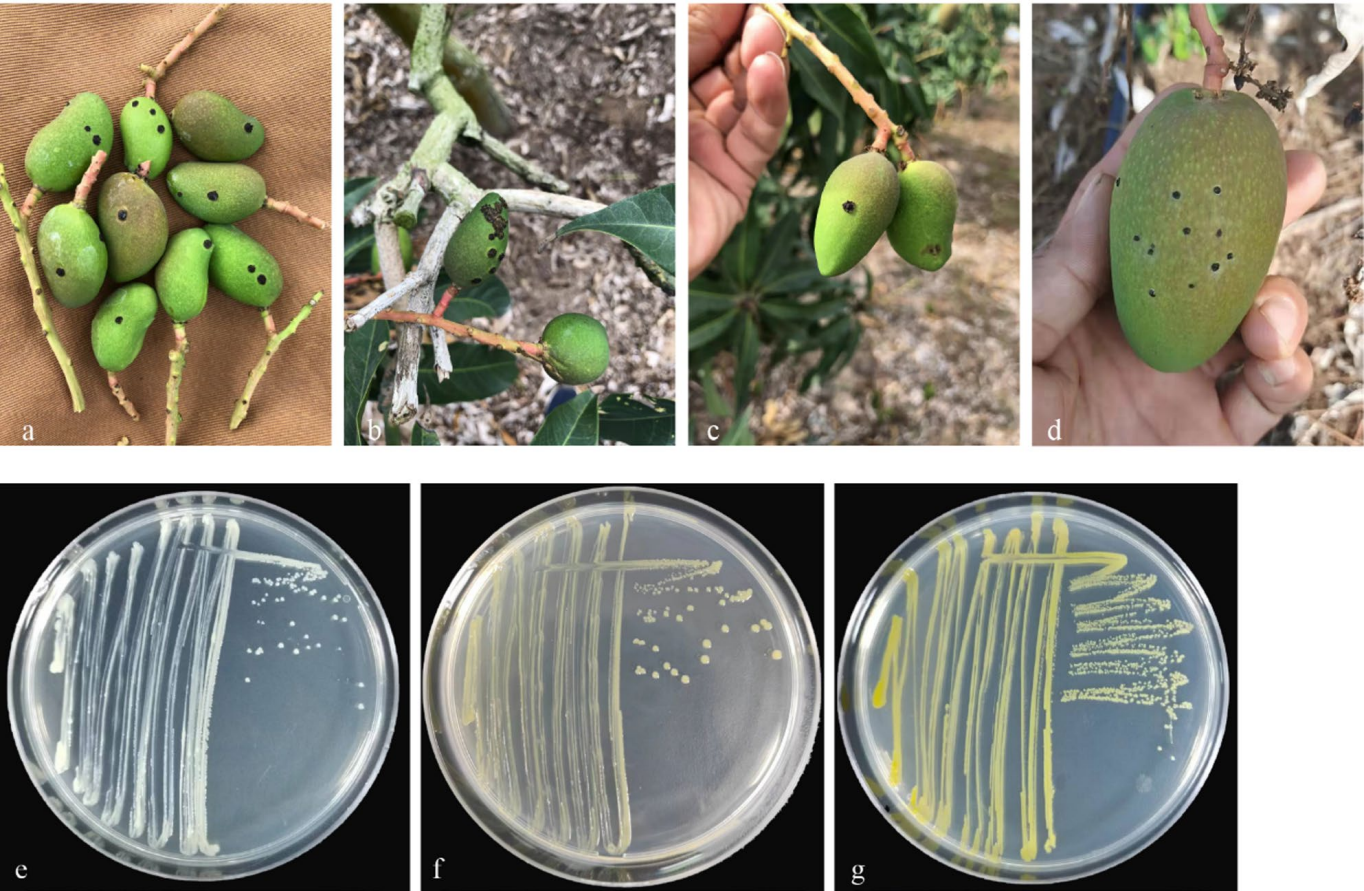
Symptoms of scab infection on mango fruit and morphology of pathogenic bacteria. **a-d** Field symptoms of mango fruit scab. **e-g** The morphology of strain MG-1, MG-2, MG-3, respectively

During field surveys in commercial mango orchards in Sanya, Hainan Province, from 2020 to 2021, diseased mango samples exhibiting scab-like symptoms similar to those caused by *Elsinoë mangiferae* were found [10] (Fig. 1a-d). In the early stage of the disease, round needle-shaped black-brown spots emerged on the surface of young fruits, resembling the symptoms of bacterial black spot and scab diseases. However, unlike in scab diseases, premature fruit drop did not occur. As the disease progressed, the surface lesions became raised, similar to the symptoms of bacterial black spot diseases. Nevertheless, in contrast to bacterial black spot, gummosis was absent. These characteristics clearly distinguish this disease from the previously reported scab and bacterial black spot diseases. In severe cases, these symptoms significantly impair the fruit’s appearance, subsequently reducing its commercial value.

### Morphological and cultural features of pathogenic bacteria

Through a series of dilution and subsequent purification procedures, three distinct colonies were isolated. Their isolation frequencies were 40%, 47%, and 13%, respectively. Notably, both white and pale-yellow colonies, which together accounted for over 40%, were the dominant strains (Table S5). Furthermore, representative strains MG-1, MG-2, and MG-3 were selected for further investigation. After incubation at 28 °C on NA agar for 24 h, all three strains produced round, translucent colonies with regular edges. The colony characteristics are as follows: MG-1 was milky white, featuring a smooth papillary surface; MG-2 was pale yellow, also with a smooth papillary surface; and MG-3 was yellow, having a raised, dry, wrinkled surface (Fig. 1e-g).

### Pathogenicity tests conducted on mango fruit and seedlings

The pathogenicity of representative strains MG-1, MG-2, and MG-3 was evaluated through indoor inoculation on both detached fruits and intact leaves. The results are summarized as follows.

**Fig. 2:**
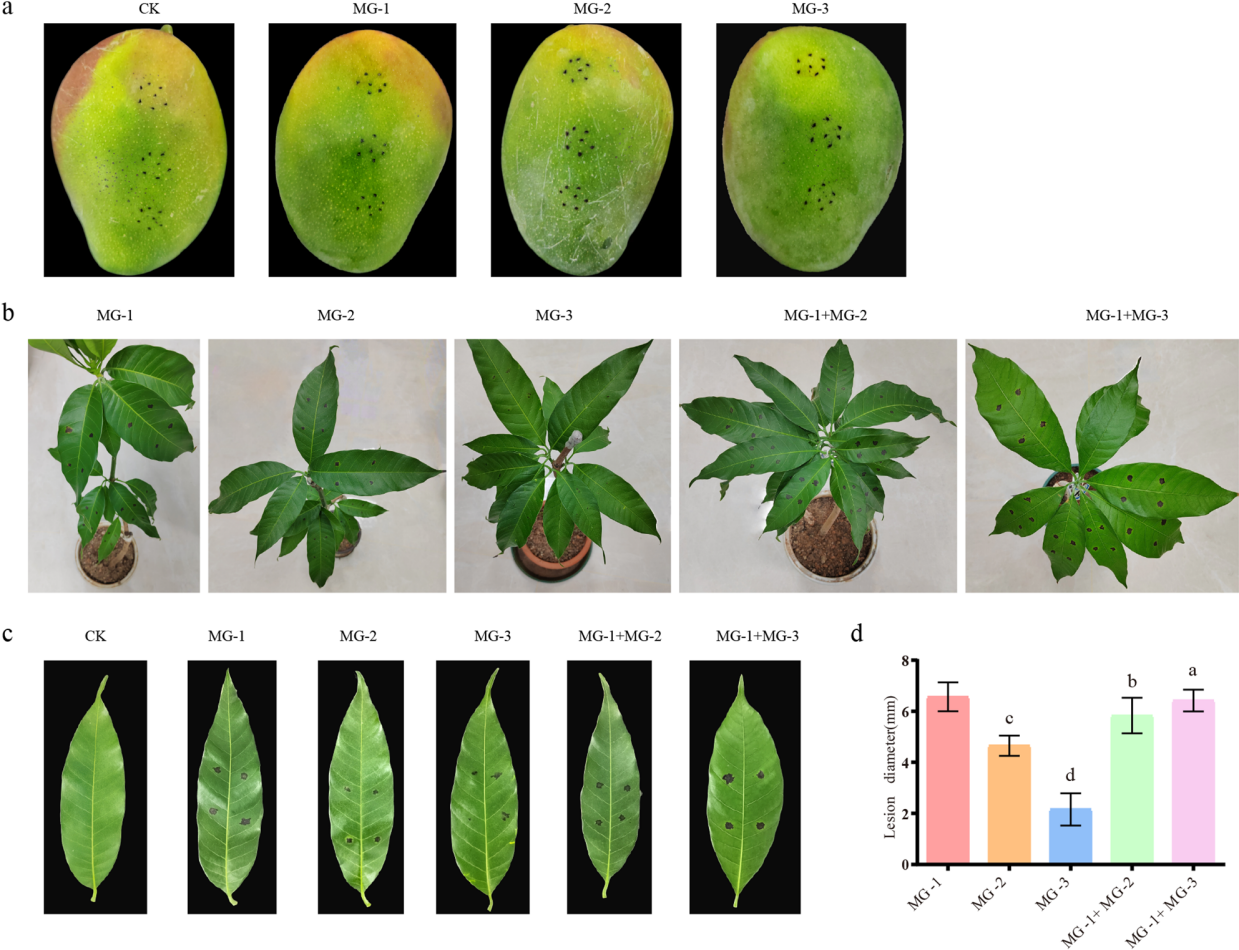
Pathogenicity assessment of the pathogen on mango fruits and leaves. **a** Symptoms resulting from artificial inoculation of mango fruit in vitro; **b-c** Artificial inoculation of mango leaves (**b**) and display of diseases on single leaves (**c**), respectively; **d** Length of lesion resulting from inoculation of mango leaves in seeding. Data are presented as the means ± SD. Different letters above columns indicate significant differences according to the Tukey test *p* ≤ 0.05

Inoculation on detached fruit: Strains MG-1, MG-2, and MG-3 were inoculated through prick wounds. The results indicated that all three strains could induce fruit pathogenesis under prick-wound inoculation conditions (Fig. 2a). The pathogenic symptoms closely resembled the early manifestations of scab. In the sterile water control group, only black areas resulting from reactive oxygen species generation were visible at the pinhole. Moreover, pathogens with morphological characteristics similar to those of the original inoculum were successfully re-isolated from the infection site.

Inoculation on live leaves: Strains MG-1, MG-2, and MG-3 were introduced into live leaves using the leaf-back pressure infiltration method. Three days after inoculation, all three strains exhibited disease symptoms at the inoculation sites. Strain MG-1 showed strong pathogenicity. When compared with single-strain MG-1 inoculation, co-inoculation with the MG-1 and MG-3 mixture showed no significant difference. In contrast, co-inoculation with the MG-1 and MG-2 mixture led to a statistically significant difference compared to single - isolate culture inoculation. As shown in Fig. 2b-d, the lesions expanded uniformly after 15 days. Notably, the lesions on the reverse side of leaves inoculated with strains MG-1 and MG-2 showed pronounced hyperplasia, while strain MG-3 did not exhibit such hyperplastic growth. The diameters of the spots caused by these three strains were 6.73 mm for strain MG-1, 4.66 mm for strain MG-2, and 2.59 mm for strain MG-3. Statistical analysis revealed significant differences among these measurements for the tested strains. The control treatment group (CK) did not develop any disease 15 days post-inoculation.

### Physiological and biochemical characterization

Based on the physiological and biochemical tests (Fig. S1; Table 1), all strains were identified as Gram-negative, motile bacteria capable of utilizing citrate and fermenting D-mannitol. However, they did not produce H_2_S or exhibit tryptophan deaminase activity. The Voges-Proskauer test results were positive, suggesting that the strains could generate pyruvate through glucose catabolism. The MG-1 strain exhibited positive urease and ornithine decarboxylase activities. It could ferment various sugars and alcohols, including D-sorbitol, melibiose, ribitol, and raffinose. Nevertheless, MG-1 tested negative for lysine decarboxylase activity, as well as for the methyl red and inositol reactions. In contrast, the MG-2 strain showed positive results for both the methyl red reaction and inositol fermentation. However, it was negative for ornithine decarboxylase and urea enzyme activities and could not ferment several sugars, such as D-sorbitol, ribitol, or raffinose, which distinguished it from the MG-1 strain. Nevertheless, both strains showed similar results for lysine decarboxylase activity and melibiose testing. The MG-3 strain was unable to ferment melibiose, ribitol, or raffinose but could ferment inositol and D-sorbitol. It produced urea but tested negative for the methyl red reaction.

**Table 1:** Physiological and biochemical characteristics of the study strains

Characteristics	MG-1	MG-2	MG-3
Motility	**+**	**+**	+
Ornithine decarboxylase	**+**	**-**	\
Lysine decarboxylase	**-**	**-**	\
Citrate utilization	**+**	**+**	+
H_2_S production	**-**	**-**	-
Urea hydrolysis	**-**	**-**	+
Tryptophan deaminase	**-**	**-**	-
Methyl red	**-**	**+**	-
Voges-Proskauer reaction	**+**	**+**	+
D-mannitol	**+**	**+**	+
Inositol	**-**	**+**	+
D-sorbitol	**+**	**-**	+
Melibiose	**+**	**+**	-
Ribitol	**+**	**-**	-
Raffinose	**+**	**-**	-

+ Positive reaction,** - **negative reaction,\ indicates not determined

### Molecular identification alongside phylogenetic analysis

**Fig. 3:**
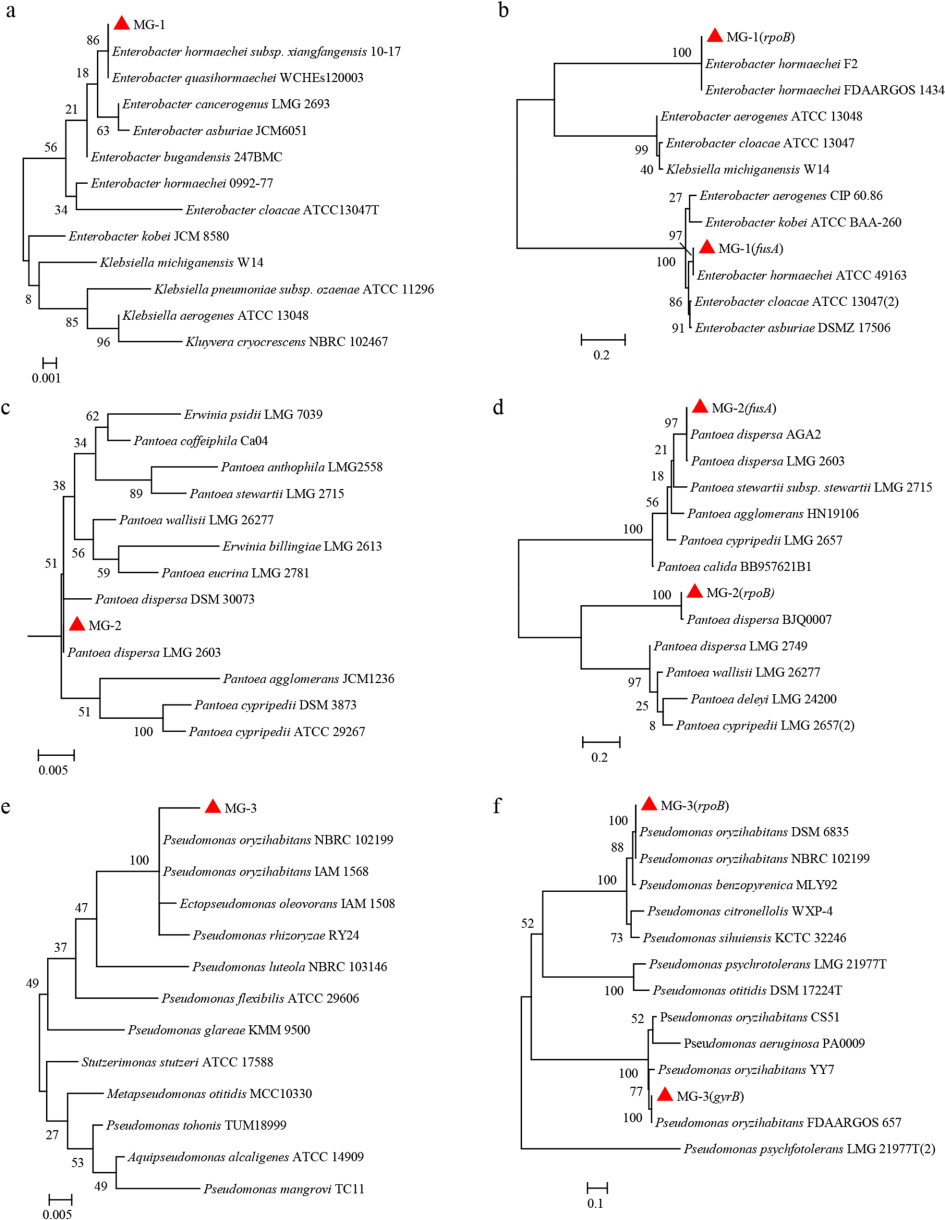
The maximum likelihood method was used to analyze of MG-1, MG-2, and MG-3. **a, c, e** maximum likelihood based on 16 S rDNA sequences of strains MG-1, MG-2, MG-3, respectively. **b, d** Phylogenetic tree of strains MG-1 and MG-2 based on maximum likelihood analyzed for the combined dataset of *fusA* and *rpoB* sequences. **f** Phylogenetic tree of strains MG-3 based on maximum likelihood analyzed for the combined dataset of *rpoB* and *gyrB* sequences. The red triangle indicates the isolates obtained in this study. Bootstrap values are indicated next to the corresponding branches

Genomic DNA was extracted from three strains (MG-1, MG-2, MG-3) and amplified using primers for 16 S rDNA, *fusA*, *rpoB*,* gyrB* (Table S1). The amplified sequences were then deposited in GenBank (accession numbers listed in Table 2). The phylogenetic tree constructed based on the 16 S rDNA gene indicated that these three strains clustered closely with the type strains of *Enterobacter* spp., *Pantoea* spp., and *Pseudomonas* spp., respectively (Fig. 3a, c, e). Notably, BLAST analysis of the 16 S rDNA gene sequences revealed similarities ranging from 99% to 100%. To further clarify the taxonomic status of these pathogenic strains, additional sequencing analyses of housekeeping genes were required.

**Table 2:** GenBank accession numbers for partial sequences of genes from three strains in the phylogenetic analysis

Strain	GenBank accession numbers			
	16 S rDNA	*rpoB*	*fusA*	*gyrB*
MG-1	OQ674725	OQ695445	OQ695447	N/A
MG-2	OQ672761	OQ695439	OQ695441	N/A
MG-3	OQ672740	OQ695451	N/A	OQ695452

“N/A” indicates no data are available in GenBank

Housekeeping gene fragments from MG-1, MG-2, and MG-3 were successfully amplified, and their sequences were submitted to GenBank (accession numbers are listed in Table 2). A maximum-likelihood tree based on *fusA* and *rpoB* gene fragments demonstrated that MG-1 clustered with several *E*. *hormaechei* strains (*E*. *hormaechei* ATCC 49163, *E*. *hormaechei* F2, *E*. *hormaechei* FDAARGOS 1434) (Fig. 3b). In the phylogenetic analyses, MG-2 clustered within the *P*. *dispersa* clade with over 90% bootstrap support (Fig. 3d). Additionally, a phylogenetic tree constructed from *rpoB* and *gyrB* gene sequences showed that strain MG-3 clustered closely with *P*. *oryzihabitans* (Fig. 3f). Therefore, strains MG-1, MG-2, and MG-3 were identified as *E*. *hormaechei*,* P*. *dispersa* and *P*. *oryzihabitans*, respectively.

“N/A” indicates no data are available in GenBank.

### Differences in extracellular enzyme and EPS production among the three strains

**Fig. 4:**
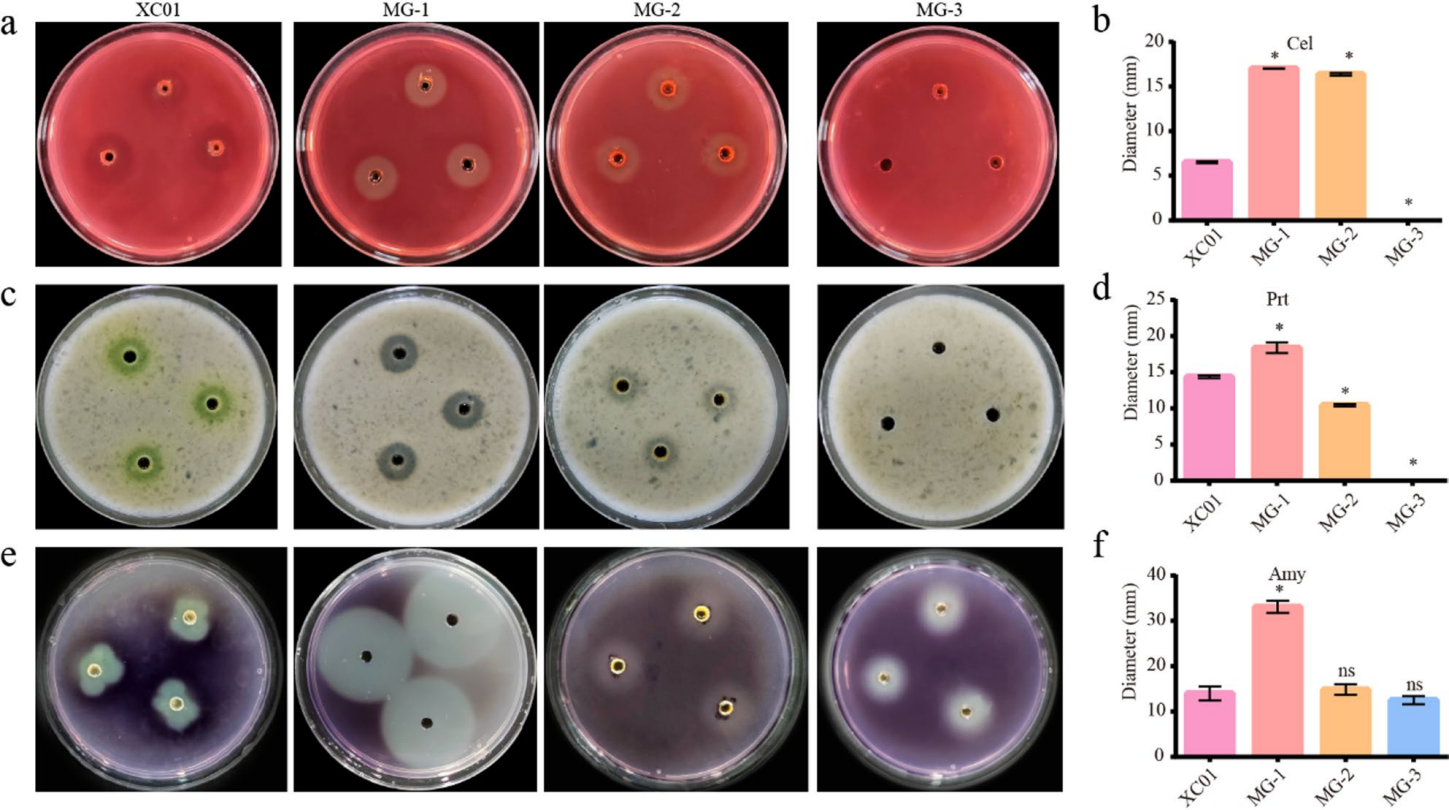
Production of virulence factors produced by strains MG-1, MG-2 and MG-3. **a** Cellulases produced by the tested strains and quantitative analysis (**b**). **c** Proteases produced by the tested strains and quantitative analysis (**d**). **e** Amylases produced by the tested strains and quantitative analysis (**f**). The data of the tested strains were compared to those of strains XC01. Data are presented as the means ± SD (*n* = 3). Statistical analyses were performed using Dunnett’s tests. * indicates *p* ≤ 0.05

To explore the pathogenicity of the isolated pathogens, the production of virulence factors, such as cellulases, proteases, and amylases, was measured. Compared with the mango bacterial leaf spot pathogen *X*. *citri* pv. *mangiferaeindicae* (XC01), strain MG-1 produced higher amounts of cellulase, protease and amylase (Fig. 4a-b). Notably, MG-2 produced more cellulases but fewer proteases, while maintaining a similar level of amylase production compared to strain XC01 (Fig. 4c-d). In contrast, MG-3 did not produce any cellulases or proteases but generated a small amount of amylase, whereas XC01 produced CWDEs (Fig. 4e-f). These results imply that *E*. *hormaechei* MG-1 and *P*. *dispersa* MG-2 may produce CWDEs to facilitate host-cell invasion, unlike *P*. *oryzihabitans* MG-3.

**Fig. 5:**
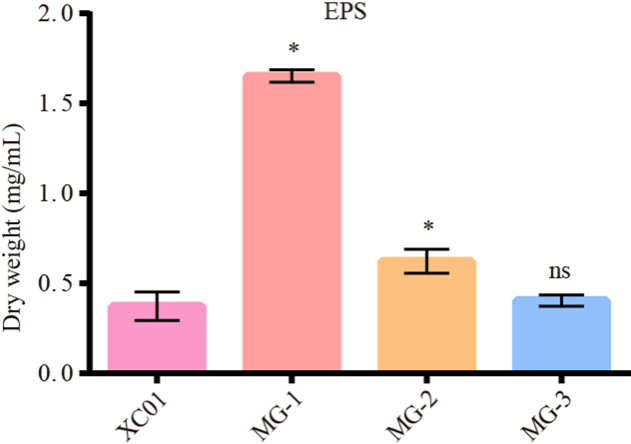
EPS produced by strains MG-1, MG-2 and MG-3. The data of the tested strains were compared to those of strains XC01. Data are presented as the means ± SD (*n* = 3). Statistical analyses were performed using Dunnett’s tests. * indicates *p* ≤ 0.05

EPS is a crucial factor contributing to the virulence of *E. asburiae* and *P. ananatis* [20]. In this study, MG-1 and MG-2 produced significantly higher amounts of EPS than XC01. Conversely, MG-3 produced a similar amount of EPS as strain XC01 (Fig. 5), suggesting that *E*. *hormaechei* MG-1 and *P*. *dispersa* MG-2 have stronger adhesive capabilities on plant surfaces than *P*. *oryzihabitans* MG-3.

### Differences in cell motility among the three strains

**Fig. 6:**
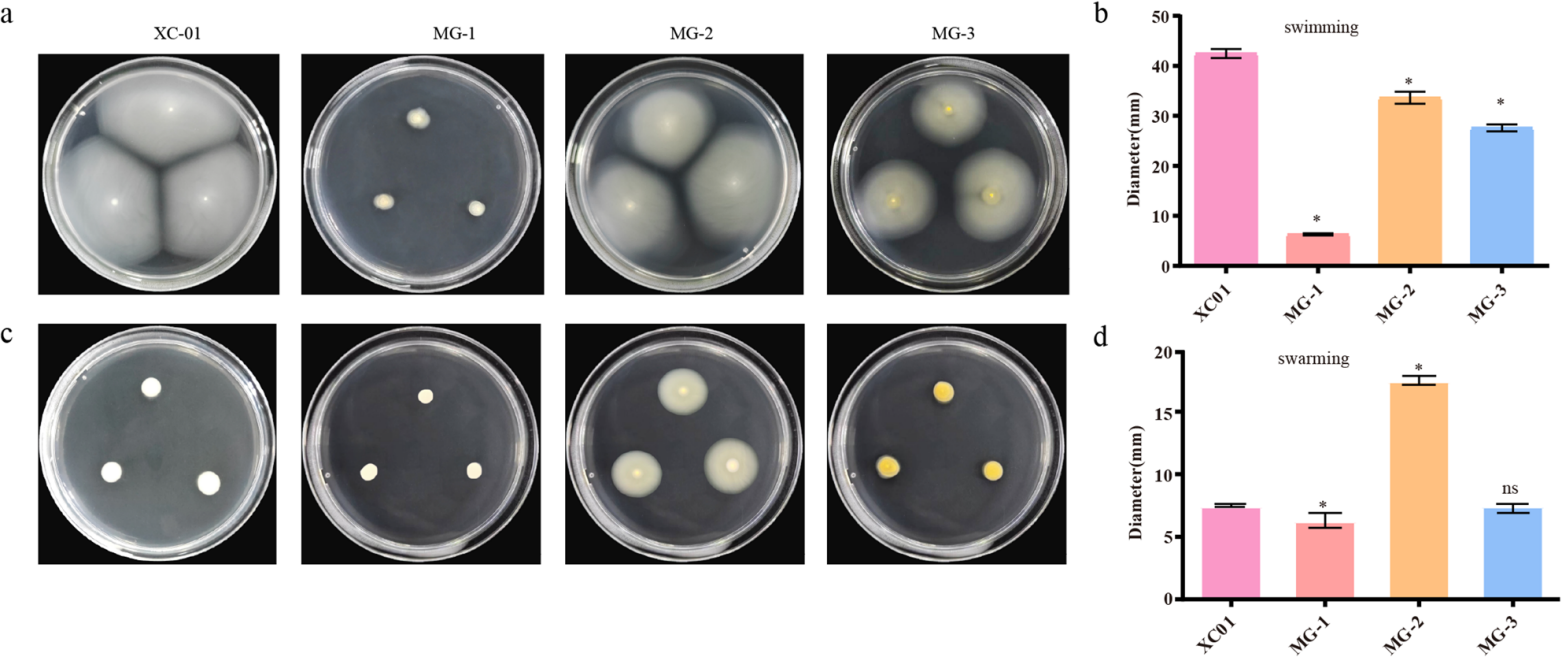
Cell motility of strains MG-1, MG-2 and MG-3. **a** Swimming motility of the tested strains and quantitative analysis (**b**). **c **Swarming motility of the tested strains and quantitative analysis (**d**). The data of the tested strains were compared to those of strains XC01. Data are presented as the means ± SD (*n* = 3). Statistical analyses were performed using Dunnett’s tests. * indicates *p* ≤ 0.05

Bacterial motility plays a crucial role in the pathogenesis during the early stages of infection [14]. The results of this study showed that some strains were capable of swimming or swarming. However, both MG-2 and MG-3 displayed lower swimming abilities than XC01(Fig. 6a-b). Notably, strain MG-2 exhibited stronger swarming behavior than XC01(Fig. 6c-d).Given that strain *P*. *oryzihabitans* MG-3 was weaker than the other two strains in production of various virulence factors and showed comparable virulence against mango leaves (Figs. 2b-d, 4 and 5), we hypothesize that the cell motility of *P*. *oryzihabitans* MG-3 may significantly contribute to its pathogenicity against mango plants, which awaits further investigations.

### Chemical agent assays

Based on the aforementioned findings (Figs. 2, 4, 5 and 6), it was concluded that strains MG-1 and MG-2 were dominant and had pronounced pathogenic properties. Consequently, twelve selected chemical agents (as described in reference 27) were evaluated. As shown in Fig. S2, Fig. S3, Table S6 and Table S7, all the tested chemical agents exhibited inhibitory effects on the growth of strains MG-1 and MG-2. Among these, four chemical agents (albendazole, tetramycin, ethylicin, bromothalonil) demonstrated significant inhibitory effects and were assessed for their efficacy against these two strains. Albendazole exhibited the most potent inhibitory effect, with EC_50_ values of approximately 0.0002 mg/L for MG-1 and 0.0001 mg/L for MG-2. Tetramycin followed, with EC_50_ values of around 0.1912 mg/L and 0.8743 mg/L, respectively. Additionally, ethylicin and bromothalonil also effectively inhibited MG-1 and MG-2, with EC_50_ values ranging from approximately 0.7497 mg/L to 15.5054 mg/L across various tested concentrations. These results indicate that albendazole is the most effective chemical agent against *E*. *hormaechei* (MG-1) and *P*. *dispersa* (MG-2), (Table 3).

**Table 3:** Inhibitory effects of various chemical agents on strain MG-1 and MG-2

Fungicide	Strain	Regression equation	Correlation coefficent	EC_50_ (mg/L)
Albendazole 20%	MG-1	y = 0.1639x + 5.5916	0.9435	0.0002
	MG-2	y = 0.1864 x + 5.7436	0.9954	0.0001
Ethylicin 80%	MG-1	y = 0.3962x + 5.2847	0.9816	0.1912
	MG-2	y = 0.3989 x + 5.0233	0.9829	0.8743
Bromothalonil 25%	MG-1	y = 0.5731x + 5.0717	0.9498	0.7497
	MG-2	y = 0.7546 x + 4.1017	0.9674	15.5054
Tetramycin 0.3%	MG-1	y = 0.4998x + 4.9353	0.9906	1.3470
	MG-2	y = 0.5706 x + 4.6687	0.9768	3.8079

## Discussion

In this study, a novel pathogen causing bacterial scab disease in mango crops in Hainan Province, China, has been identified. *Elsinoë mangiferae* is known as the causative agent of mango scab disease in Australia, the United States of America, and several countries in Africa and Asia, leading to significant losses in marketable fruit yields [10]. The symptoms caused by this newly-identified pathogen closely resemble those induced by *Elsinoë mangiferae*. Both pathogens primarily affect fruits in the field and can also impact leaves through indoor inoculation. The milky-white, pale-yellow, and yellow colonies isolated from diseased mango tissues from commercial orchards over different years were identified as *E*. *hormaechei*, *P*. *dispersa*, and *P*. *oryzihabitans* through phylogenetic analysis and physiological and biochemical characterization [42–44].

An increasing number of plant diseases caused by multiple pathogens have been reported. For instance, *E. asburiae* SC1 and *P. ananatis* SC7 induce rice bacterial blight and can cause analogous symptoms on rice leaves, seedlings, and banana plants [20]. *Citrobacter braakii* and *E. hormaechei* are emerging plant pathogens associated with walnut decline, characterized by necrotic lesions with blackened tissue [45]. In our study, we found that *E*. *hormaeche*i, *P*. *dispersa*, and *P*. *oryzihabitans* were isolated from the same tissue samples and contributed to the onset of mango bacterial scab disease. Moreover, it was determined that *E*. *hormaeche*i and *P*. *dispersa* displayed stronger pathogenicity than *P*. *oryzihabitans*. However, no co-inoculation experiments with mixtures of these three strains were conducted in this study. Future research should explore potential synergistic or inhibitory interactions among these strains.

*Gibbsiella quercinecans* and *Rahnella victoriana* were isolated from walnut trees showing symptoms of canker and stem-tissue necrosis. The pathogenicity of these strains is attributed to the production of plant CWDEs, such as cellulase [45, 46]. *Rhizoctonia solani* causes peanut sheath blight, with symptoms of tissue maceration and necrosis mainly due to CWDEs [47]. Previous studies have shown that *E. asburiae* and *P. ananatis* can produce cellulases, proteases and EPS [14, 20, 39]. Our further analysis revealed that *E*. *hormaeche*i and *P*. *dispersa* can synthesize cellulases, proteases, and amylases, whereas *P*. *oryzihabitans* cannot produce cellulases and proteases. Notably, *E*. *hormaeche*i and *P*. *dispersa* produced significantly higher amounts of EPS than strain XC01. These results may account for why *E*. *hormaeche*i and *P*. *dispersa* exhibit greater virulence towards mangoes, which is consistent with the observed symptoms on live leaves.

In this study, *E*. *hormaeche*i demonstrated significantly lower swimming and swarming motility abilities compared to other strains. This suggests that its pathogenicity may be more closely associated with CWDEs and EPS rather than motility. Although several genomes and virulence factors of *E*. *hormaeche*i and *P*. *dispersa* linked to human infections have been published, there is a scarcity of reports regarding the genomes of these species in the context of plant diseases [48–51]. Genome sequencing of these strains was not carried out in this study. Therefore, we plan to conduct further investigations on the gene clusters influencing the virulence factors of both *E*. *hormaeche*i and *P*. *dispersa*.

Additionally, it has been reported that *P*. *oryzihabitans* produces indole-3-acetic acid (IAA), which promotes plant growth and alters root bacterial community structures by increasing the diversity and abundance of beneficial taxa [52]– [53]. Moreover, this bacterium has been found to cause diseases in muskmelon, peppers and walnuts, characterized by symptoms such as soft rot, necrosis, browning, and wilting [16, 44, 54]. In this study, we found that *P*. *oryzihabitans* could also induce black spots at the inoculation sites on mango fruits and leaves, but it did not cause hyperplastic growth on the leaves. Pauwelyn et al. [23] reported that cichofactins of *Pseudomonas cichorii* SF1-54 affect swarming motility and biofilm formation through the encoding of *cifA* and *cifB* genes; Crawford et al. [55] identified (S)-valdiazen, an auto-inducing small-molecule signal in *Pseudomonas entomophila* that regulates bacterial cell-to-cell communication and virulence traits. Further analysis showed that *P*. *oryzihabitans* has lower swimming and swarming motility abilities than strain XC01. We hypothesize that the motility of *P*. *oryzihabitans* is related to its virulence. Future studies should focus on exploring the virulence mechanisms of *P*. *oryzihabitans*.

In the chemical agent test, the EC50 values of albendazole were found to be ≤ 0.01 mg/L, whereas those of three other chemical agents were ≥ 0.1 mg/L. Results from the indoor toxicity tests indicated that albendazole was the most effective agent in controlling *E*. *hormaeche*i and *P*. *dispersa*. It has been reported that albendazole (ABZ) directly binds to and inhibits aspartate aminotransferase FocAST2. It disrupts infection-related morphogenesis, and suppresses the growth of *Fusarium oxysporum* f. sp. *cubense* Tropical Race 4 [56]. It is hypothesized that genes encoding aminotransferase homologous sequences in bacteria may also serve as potential targets for albendazole, thereby inhibiting bacterial proliferation. Therefore, further research on the control efficacy of albendazole against bacterial scab disease of mango in the field is necessary. Ethylicin came next, with EC_50_ values of 0.1912 mg/L for *E*. *hormaeche*i and 0.8743 mg/L for *P*. *dispersa*. These values deviated from previous research findings, and this discrepancy may be attributed to differences in host organisms [57]. Tetramycin exhibited strong activity against a broad spectrum of fungi and effectively controls *Colletotrichum scovillei* on detached pepper fruits [58]. In this study, tetramycin and bromoxanil also effectively controlled *E*. *hormaeche*i and *P*. *dispersa*. These findings provide significant insights into field control strategies for managing *E*. *hormaeche*i and *P*. *dispersa*.

## Conclusions

In summary, this study is the first to report that *E*. *hormaeche*i, *P*. *dispersa*, and *P*. *oryzihabitans* are plant pathogens causing mango bacterial scab in Hainan Province of China. We have elucidated their biological and pathological characteristics and identified effective chemical agents against these pathogens in this region. The findings of this study can serve as a basis for the sustainable management of mango bacterial scab caused by these bacterial agents. They also facilitate informed decision-making for future disease control strategies.

## Supplementary Information

Additional file 1: Fig. S1. Gram staining results of pathogenic bacteria. Fig. S2. The results of medicament test on LB medium of MG-1 strain. Fig. S3. The results of medicament test on LB medium of MG-2 strain. Table S6. The results of medicament test on LB medium of MG-1 strain. Table S7. The results of medicament test on LB medium of MG-2 strain. 

Additional file 2: Supplementary Information Additional file 2: Table S1. Primers used in this study. Table S2. GenBank accession number of strains used in this study. Table S3. The media used in this study. Table S4. Concentrations of substances used for fungicide sensitivity assays. Table S5. Isolation rates of three types of colonies in infected fruits.

## Data Availability

All data and material are available upon request to correspondence author. All data has already been deposited in the National Center for Biotechnology Information (NCBI) database (www.ncbi.nlm.nih.gov/search/), and were assigned the accession numbers that list in Table 2 and Additional file 2: Table S2). Additionally, the accession numbers of the taxa newly described in this study (*Enterobacter hormaechei* MG-1: 16 S rDNA, OQ674725; *rpoB*, OQ695445; *fusA*, OQ695447; *Pantoea dispersa* MG-2: 16 S rDNA, OQ672761; *rpoB*, OQ695439; *fusA*, OQ695441; *Pseudomonas oryzihabitans* MG-3: 16 S rDNA, OQ672740; *rpoB*, OQ695451; *gyrB*, OQ695452 ) are shown in Table 2.

## Abbreviations

E. hormaechei: Enterobacter hormaechei
P. dispersa: Pantoea dispersa
P. oryzihabitans: Pseudomonas oryzihabitans
X. citri pv. mangiferaeindicae: Xanthomonas citri pv. Mangiferaeindicae
EPS: extracellular exopolysaccharides
EC_50_: the concentration required for 50% maximal effect
rpoB: RNA polymerase β subunit
fusA: elongation factor G
gyrB: DNA gyrase subunit B
LB: luria-bertani
NA: nutrient agar
CWDEs: cell wall degrading enzymes
E. asburiae: Enterobacter asburiae
P. ananatis: Pantoea ananatis
