# Supplementary material for: Identification, pathogenicity, and virulence of the causal agent of bacterial scab disease on mango in China
Source: BMC Microbiol. 2025 Dec 22;25:808. doi: 10.1186/s12866-025-04656-3 (PMC12752356; doi:10.1186/s12866-025-04656-3)
Supplement: Supplementary file 1 — Additional file 1: Fig. S1. Gram staining results of pathogenic bacteria. Fig. S2. The results of medicament test on LB medium of MG-1 strain. Fig. S3. The results of medicament test on LB medium of MG-2 strain. Table S6. The results of medicament test on LB medium of MG-1 strain. Table S7. The results of medicament test on LB medium of MG-2 strain. [file 12866_2025_4656_MOESM1_ESM.docx]

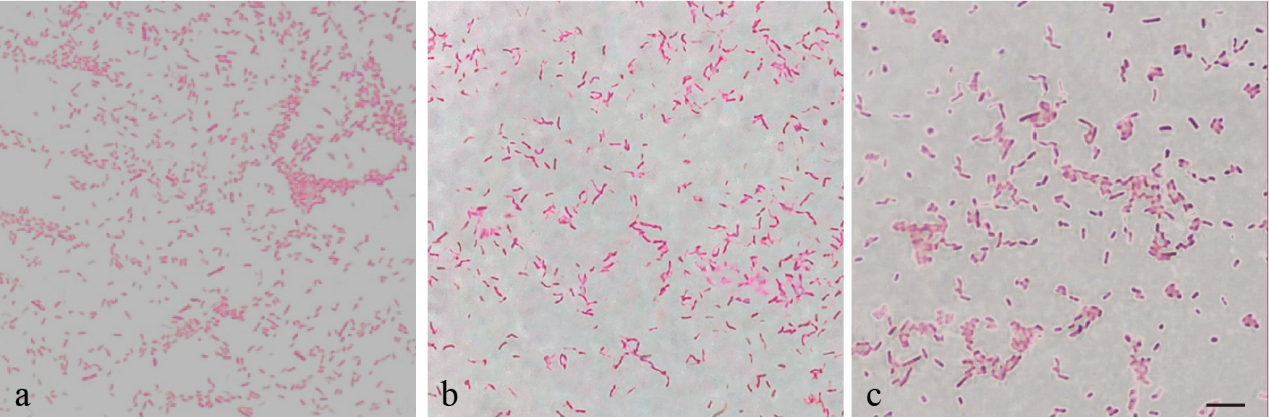


**Fig. S1** Gram staining results of pathogenic bacteria. (a-c) are designated as MG-1, MG-2, MG-3, respectively. Scale bars =10 μm.


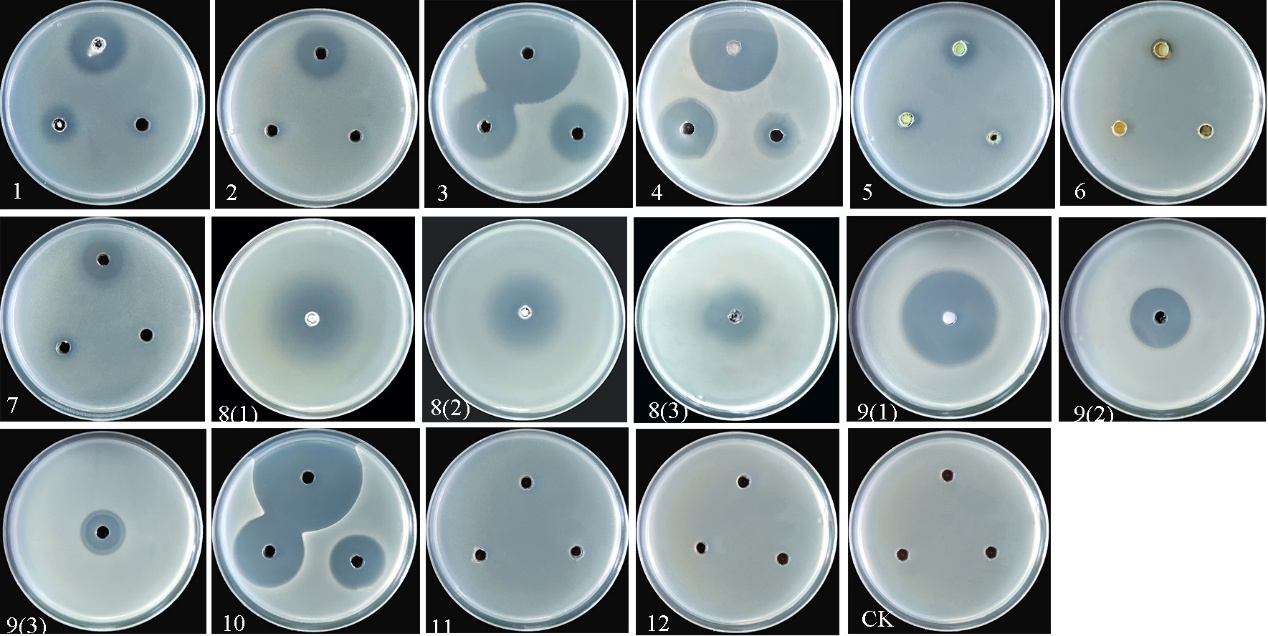


**Fig. S2** The results of pharmaceutical test on LB medium of MG1 strain. In the figure, numbers 1, 2, 3, 4, 5, 6, 7, 10, 11 and 12 respectively represent Zinc thiazole suspension agent, Kasugamycin, Benziothiazolinone, Bromothalonil, Oxine-copper, Thiosen copper, Cuppric nonylphenolsulfonate, Tetramycin, Mesomycin and Copper hydroxide, which are consistent with the numbers of the tested fungicide. 8 (1), 8 (2) and 8 (3) represent the 100 mg·L-1, 10 mg·L-1 and 2 mg·L-1 dilutions of Albendazole respectively; 9 (1), 9 (2) and 9 (3) respectively represent the 100 mg·L-1, 10 mg·L-1 and 2 mg·L-1 dilutions of Ethylicin. CK is the LB medium carrying bacteria. 40 μL of sterile water is added to each of the three dosing holes. For other culture dishes carrying bacteria, add 40 μL of the fungicide at concentrations of 100 mg·L-1, 10 mg·L-1 and 2 mg·L-1 in a clockwise direction from the first hole on the right side to the dosing holes in sequence.


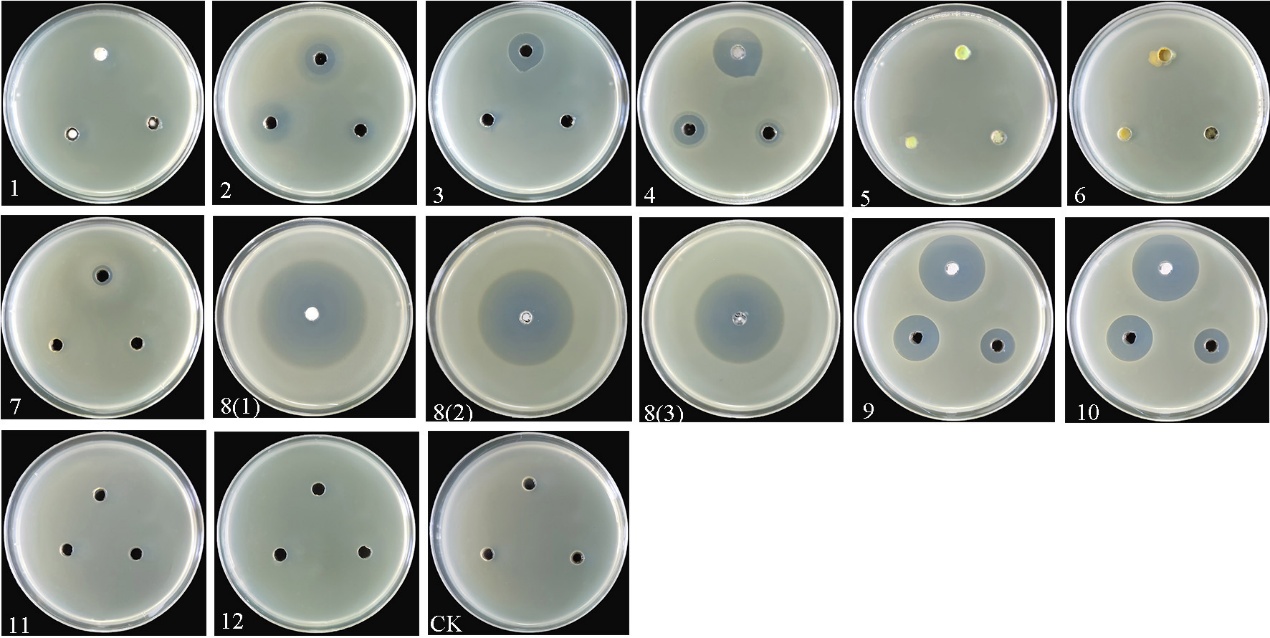


**Fig. S3** The results of pharmaceutical test on LB medium of MG2 strain. In the figure, numbers 1, 2, 3, 4, 5, 6, 7, 9, 10, 11 and 12 respectively represent Zinc thiazole suspension agent, Kasugamycin, Benziothiazolinone, Bromothalonil, Oxine-copper, Thiosen copper, Cuppric nonylphenolsulfonate, Ethylicin, Tetramycin, Mesomycin and Copper hydroxide, which are consistent with the numbers of the test reagents. 8 (1), 8 (2) and 8 (3) represent the 100 mg·L-1, 10 mg·L-1 and 2 mg·L-1 dilutions of Albendazole respectively; CK is the LB medium carrying bacteria. 40 μL of sterile water is added to each of the three dosing holes. For other culture dishes carrying bacteria, add 40 μL of chemicals with concentrations of 100 mg·L-1, 10 mg·L-1 and 2 mg·L-1 in a clockwise direction from the first hole on the right side to the dosing holes in sequence.

**Table S6** The results of medicament test on LB medium of MG-1 strain

| Number | Fungicide | Diameter of suppression（mm）^a^ | | |
| --- | --- | --- | --- | --- |
|  |  | 100 mg·L^-1^ | 10 mg·L^-1^ | 2 mg·L^-1^ |
| 1 | Zinc thiazole suspension agent 40% | 6.36±0.39 | 0 | 0 |
| 2 | Kasugamycin 4% | 18.57±0.47 | 0 | 0 |
| 3 | Benziothiazolinone 3% | 22.58±0.66 | 8.40±0.41 | 6.14±0.27 |
| 4 | Bromothalonil 25% | 43.57±0.89 | 20.32±1.38 | 4.82±0.60 |
| 5 | Oxine-copper 33.5% | 2.56±0.33 | 0 | 0 |
| 6 | Thiosen copper 30% | 3.40±0.18 | 0 | 0 |
| 7 | Cuppric nonylphenolsulfonate 30% | 11.74±0.28 | 1.90±0.31 | 0 |
| 8 | Albendazole 20% | 38.50±0.18 | 31.35±1.77 | 24.60±0.58 |
| 9 | Ethylicin 80% | 52.13±0.13 | 33.79±0.10 | 21.51±0.23 |
| 10 | Tetramycin 0.3% | 43.56±0.15 | 26.56±0.30 | 23.51±0.25 |
| 11 | Mesomycin 3% | 0 | 0 | 0 |
| 12 | Copper hydroxide 46% | 0 | 0 | 0 |

^a^Bacteriostatic diameter = average inhibition circle diameter -7 mm (diameter of hole) ± standard deviation.

**Table S7** The results of pharmaceutical test of MG-2 on LB medium

| Number | Fungicide | Diameter of suppression（mm）^a^ | | |
| --- | --- | --- | --- | --- |
|  |  | 100 mg·L^-1^ | 10 mg·L^-1^ | 2 mg·L^-1^ |
| 1 | Zinc thiazole suspension agent 40% | 0 | 0 | 0 |
| 2 | Kasugamycin 4% | 14.31±0.50 | 4.07±0.04 | 1.38±2.40 |
| 3 | Benziothiazolinone 3% | 8.75±0.94 | 1.21±0.10 | 0 |
| 4 | Bromothalonil 25% | 16.97±0.81 | 10.52±0.46 | 5.75±0.11 |
| 5 | Oxine-copper 33.5% | 0 | 0 | 0 |
| 6 | Thiosen copper 30% | 0 | 0 | 0 |
| 7 | Cuppric nonylphenolsulfonate 30% | 2.56±0.17 | 0 | 0 |
| 8 | Albendazole 20% | 47.63±1.56 | 38.12±0.73 | 35.72±0.34 |
| 9 | Ethylicin 80% | 24.71±0.38 | 15.27±0.22 | 10.30±0.18 |
| 10 | Tetramycin 0.3% | 18.88±0.31 | 9.63±0.34 | 6.04±0.05 |
| 11 | Mesomycin 3% | 0 | 0 | 0 |
| 12 | Copper hydroxide 46% | 0 | 0 | 0 |

^a^Bacteriostatic diameter = average inhibition circle diameter -7 mm (diameter of hole) ± standard deviation.
